# Intra-Decadal (2012–2021) Dynamics of Spatial Ichthyoplankton Distribution in Sevastopol Bay (Black Sea) Affected by Hydrometeorological Factors

**DOI:** 10.3390/ani12233317

**Published:** 2022-11-28

**Authors:** Marina Basova, Svetlana Krasheninnikova, Vincenzo Parrino

**Affiliations:** 1 Moscow Representative Office, A.O. Kovalevsky Institute of Biology of the Southern Seas of Russian Academy of Sciences (IBSS RAS), Leninskiy Ave. 38/3, 119991 Moscow, Russia; 2 Department of Chemical, Biological, Pharmaceutical, and Environmental Sciences, University of Messina, Viale Ferdinando Stagno d’Alcontres 31, 98166 Messina, Italy

**Keywords:** eggs, larvae, ichthyoplankton abundance, temperature, wind, interannual variability, intra-decadal variability, pollution, monitoring

## Abstract

**Simple Summary:**

Due to the high mortality of fish in the early stages of ontogenesis, the detection of factors affecting it seems to be an extremely important problem. We investigated intra-decadal dynamics and spatial distribution of the ichthyoplankton abundance that depends on hydrometeorological factors. We focused on Sevastopol Bay and its four areas. The ichthyoplankton distribution by areas of Sevastopol Bay was associated with the peculiarities of the pollution distribution and with the wind regime. For the first time, a positive effect of temperature and repeatability of northern and northeastern winds on the ichthyoplankton number during the summer spawning in all areas was reported. The abundance of ichthyoplankton is influenced by several factors: (a) In the southern area, a positive influence of temperature and northerly winds on the ichthyoplankton abundance was confirmed. (b) In the eastern area, the discharge of rivers is an additional factor. (c) Characteristics of winds in western and central areas are significantly impacted by the orography peculiarities. The data obtained during the research highlighted the importance of comprehensive monitoring studies using an intra-decadal approach on scrutinizing ichthyoplankton abundance, distribution and many other characteristics.

**Abstract:**

High mortality of fish in the early stages of ontogenesis requires the detection of factors affecting it and is an extremely important problem. For this reason, we tried to consider the influence of some hydrometeorological factors on the intra-decadal dynamics and spatial distribution of ichthyoplankton abundance in Sevastopol Bay. To this end, we analyzed ichthyoplankton samples collected in 2012–2021 in Sevastopol Bay, and the four districts were identified. The ichthyoplankton distribution by areas was associated with the peculiarities of the pollution distribution. The maximum numbers in eggs (34.7 ± 7.5 ind. m^−2^) and larvae (10.0 ± 2.4 ind. m^−2^) were recorded in July–August. For the first time, a positive effect of temperature and repeatability of north and northeast winds on the ichthyoplankton number during the summer spawning in all identified areas of Sevastopol Bay using principal component analysis (PCA) was reported. In the southern region, positive influence of temperature and northerly winds on the ichthyoplankton abundance was confirmed by ANOVA (*p* = 0.02), and Pearson’s correlation analysis revealed significant correlations between these parameters on inter-annual scale (r > 0.5). In the eastern area, the discharge of rivers is an additional factor affecting the abundance of ichthyoplankton, while in the western and central areas, these factors were the wind characteristics caused by the orography peculiarities. The pollution spread in Sevastopol Bay is also largely due to the wind regime. The importance of comprehensive monitoring studies using intra-decadal data on ichthyoplankton is emphasized.

## 1. Introduction

One of the fundamental sections of fish population dynamics problem is the question of survival in their early stages of development. The eggs and larvae survival in the early stages of ontogenesis determines the final replenishment of fish populations. In this regard, a significant place in marine ichthyology is given to the study of planktonic stages of fish development [1]. For this reason, the quantitative accounting of ichthyoplankton is considered as one of the most representative methods of estimating the fish population number. In the Black Sea, as in other seas of the temperate zone with well-defined seasonality, the timing of fish spawning by the temperature regime is largely determined. The variability of water temperature leads to a species change composition and fish eggs and larvae abundance [2].

**Figure 1:**
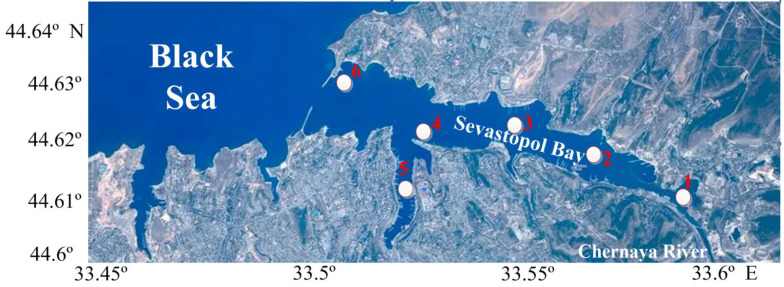
Map showing the study area of ichthyoplankton sampling stations in Sevastopol Bay: 1, Inkerman; 2, Sukharnaya bay; 3, Avlita; 4, Pavlovsky Cape; 5, South Bay; 6, Ravelin.

Depending on the time of year and species features, spawning areas can be a narrow 1.5, 2, 10-mile zone, pre-watering, estuarine areas, etc. [2]. These areas are important habitats and spawning grounds for many permanent and migratory marine fish species. Thus, study on the ichthyoplankton complexes in closed and partially closed basins is of particular interest. Such a basin is the unique and size significant semi-enclosed estuary-type water area stretched from east to west the Sevastopol Bay. The interannual variability of the ichthyoplankton abundance in different areas of the Sevastopol Bay and its determining factors had been studied very fragmentally [3,4,5,6,7,8,9]. At the same time, significant data on the hydrodynamic and hydrochemical regimes of the bay have been collected [10,11,12,13,14,15]. Some previous works investigated the causes of pollution and their spatial distribution over the water area of the Sevastopol Bay [13,16]. The coastline of the Sevastopol Bay is characterized by significant indentation, due to the presence of bays and capes (Figure 1), that affects the ichthyoplankton spatial distribution. Seasonal and interannual dynamics of ichthyoplankton abundance and their dependence on the strength and repeatability of winds at individual stations inside the Sevastopol Bay have not been practically studied [13,17]. It is well known that ichthyoplankton, being basically a passive form, is distributed over the water area under the influence of dynamic factors, which include wind effect [2]. Thus, continuous monitoring of the dynamics of ichthyoplankton abundance along at separate stations of the Sevastopol Bay and its influence of hydrometeorological environmental factors is required. These data allow assessment and prediction of favorable conditions for the development of eggs and juvenile fish within the study area and make recommendations necessary for the preservation of spawning grounds and fish stocks in the region under the threat of the climate changes.

We set out to address the following questions: (1) Are wind characteristics and water temperature related to the distribution of ichthyoplankton on a very significant area of Sevastopol Bay? (2) Are there any patterns influencing these factors on the abundance of ichthyoplankton throughout the year and on an inter-annual and intra-decadal scale?

## 2. Materials and Methods

### 2.1. Study Area

To analyze intra-annual and inter-annual variability, as well as the spatial distribution of fish eggs and larvae abundance in the Sevastopol Bay, data collected from six different stations along the bay (Figure 1) during 2012–2021 were used.

Sevastopol Bay is a narrow and non–freezing semi-enclosed estuarine water area in the Black Sea. The bay extends into the southwestern part of the Crimean Peninsula for 7.5 km with a maximum width of about 1 km. It is located to the east between the southern and northern protective piers, protecting it from wind, waves and swell. The eastern tip of Bay is the mouth of the Chernaya River. The Bay area is 7.96 km^2^ with maximum depth 21 m. Average depth is 12.5 m. The eastern tip of Bay is the mouth of the Chernaya River with length 35.0 km, catchment area 427 km^2^ and river slope 8.6 m/km. Before the mouth, two right tributaries flow into the Black River, one of which (Aitodorka) is of sufficient importance, since it is fed by springs, and the other (Suchaya River) brings rainwater to the river. The Mediterranean type of climate in the Sevastopol area determines the seasonal nature of precipitation distribution. This climate is characterized by winter precipitation maxima of up to 50%, and summer minima of about 15–20%. Local circulation also does not contribute to precipitation, as well as high insolation characteristics of coastal zones. Thus, the seasonality of precipitation in the bay area is determined by the type of climate, the circulation features of the terrain and the features of orography.

### 2.2. Field Sampling and Processing

We caught ichthyoplankton up to three times a month during 2012–2021. To do this, small vessels followed the entire water area of the Sevastopol Bay, but at specific stations. Ichthyoplankton samples were collected at 6 stations above a depth of 6 to 20 m. The collection of ichthyoplankton was carried out by a Bogorov-Rass network (500 μm cells, 0.5 m^−2^ inlet area) and Duday network (500 μm cells, the area of the inlet 0.36 m^−2^). The samples were fixed with a 4% formaldehyde solution and then processed in laboratory conditions. Simultaneously measurements of water temperature (T °C), as well as strength (W, in points) and wind directions (8 directions: N, NE, NW, S, SW, W, SE, E) were made. The wind repeatability of different directions was calculated as a percentage of the total number of events during the observation period under consideration.

### 2.3. Data Analysis

According to these data, the calculation of the average values for the parameters under consideration and their standard deviations characterizing the inter-annual variability was carried out. Based on the assessment of linear trends, the analysis of intra–decadal trends of the ichthyoplankton number and hydrometeorological parameters for 2012–2021 was carried out. The significance of trends at the 95% confidence level by the value of the coefficient of determination (R2) exceeding the threshold value of 0.4 was estimated. This value R2 characterizes the contribution of the variance introduced by the trend to the overall variance.

Principal component analysis (PCA) was applied to identify the most significant factors explaining the means of eggs and larvae. Mean values, standard deviations (±SD) and Pearson’s correlation coefficients (r) using Microsoft Excel 2010 (https://statpsy.ru/pearson) (accessed on 23 February 2022) were calculated. Confidence levels (*p*) for the correlation coefficients were assessed according to Müller et al. [18]. The significance of linear trends was tested using the Student’s *t*-test [19]. To identify a reliable cumulative effect of the influence of hydrometeorological environmental factors on the intra-decadal variability and spatial distribution of the ichthyoplankton abundance, multiple regression analysis of ANOVA was performed.

## 3. Results

### 3.1. Intra-Annual Variability of Ichthyoplankton Abundance

**Figure 2:**
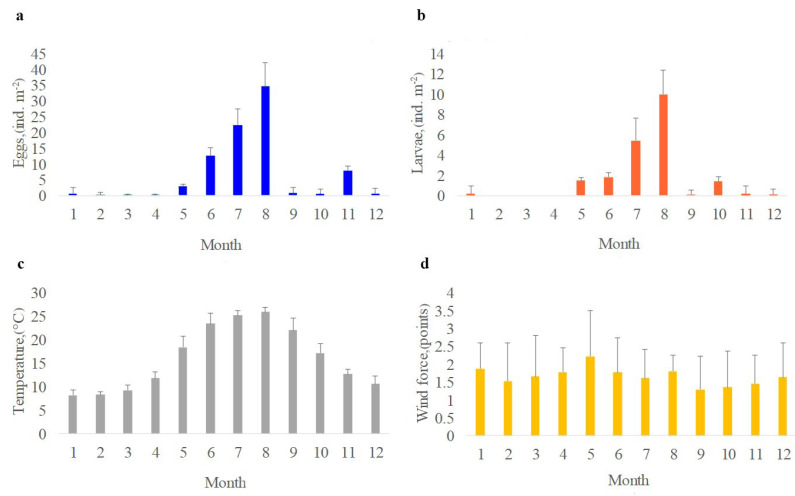
Intra-annual variability of the average values for the number of eggs (**a**), larvae (**b**), water temperature (**c**), the strength of the dominant north wind (**d**) with standard deviations in Sevastopol Bay in 2012–2021.

The intra–annual variability of the average values for the ichthyoplankton abundance and hydrometeorological characteristics for the Sevastopol Bay for 2012–2021 is shown in Figure 2. Maximum numbers of eggs 34.7 ± 7.5 ind. m^−2^ and larvae 10.0 ± 2.4 ind. m^−2^ were reached in the summer (July–August), whereas the minimum eggs 0.1 ± 0.05~0 ind. m^−2^ and larvae 0.0, respectively in March–April were found, respectively (Figure 2a,b). As expected, the highest temperatures are observed in summer −26.0 ± 1.0 °C in August and the minimum—in winter −8.2 ± 0.9 °C in January–February (Figure 2c).

We investigated the number of wind phenomena (eight directions) during the year for the period 2012–2021 at each station and in the entire Sevastopol Bay as a whole, synchronously with the ichthyoplankton samples selection. In addition, the calculated repeatability for the winds in the different directions was also analyzed.

As a result of the data analysis, winds of the north, south-west and south directions dominated in the Sevastopol Bay, and the repeatability was equal to 34, 19 and 14%, respectively (Figure 2d). The wind strength of the dominant northern direction was the lowest in autumn −1.29 ± 0.8 points, and the highest in May −2.2 ± 0.6 points.

**Figure 3:**
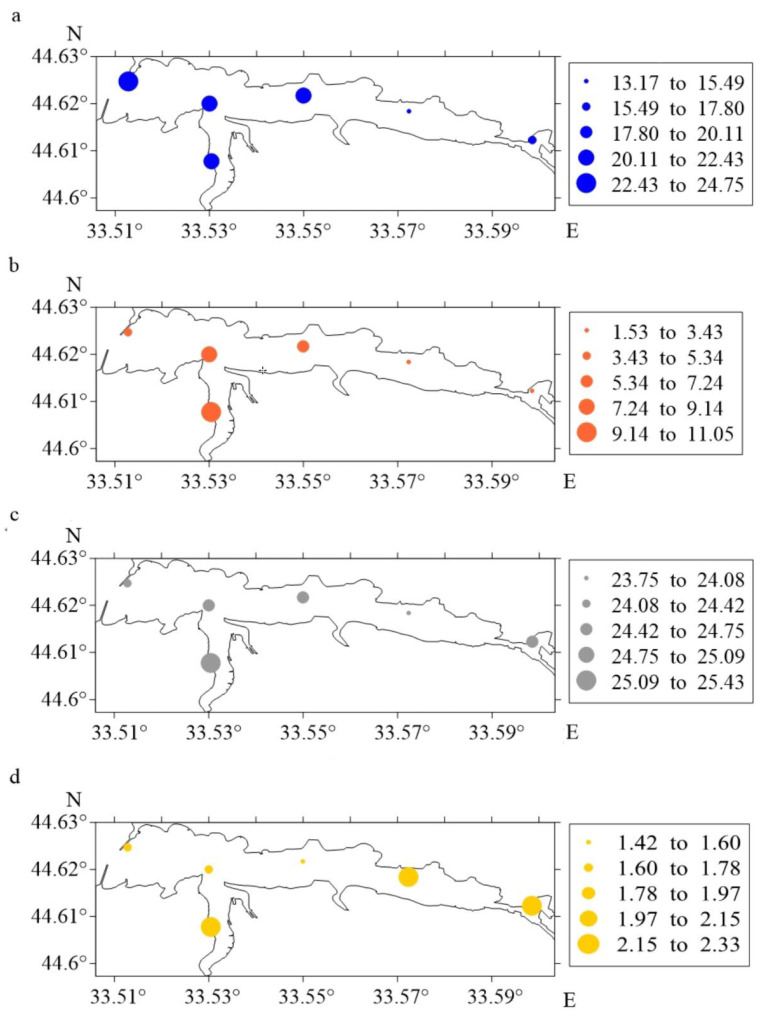
The number of eggs ((**a**), ind. m^−2^), larvae ((**b**), ind. m^−2^), as well as the water temperature ((**c**), °C) and the strength of the wind ((**d**), points) in the Sevastopol Bay in the summer of 2012–2021.

During summer spawning, the maximum number of eggs and larvae was noted (Figure 2a,b). Figure 3 and Table 1 report the distribution of ichthyoplankton, water temperature and wind strength indicators in the summer season in Sevastopol Bay during 2012–2021.

The average values for the ichthyoplankton abundance and hydrometeorological characteristics and their standard deviations in the Sevastopol Bay in the summer of 2012–2021 are shown in Table 1.

The maximum number of eggs during the spawning at the Ravelin station was 24.74 ± 4.95 ind. m^−2^. Values close to maximum were obtained at the Pavlovsky Cape, South Bay and Avlita stations. The lowest values for the indicator were marked at the Sukharnaya and Inkerman stations (13.17 ± 2.63 and 16.06 ± 3.21 ind.m^−2^). The highest larvae concentration was recorded at the Southern Bay on 11.04 ± 2.21 ind.m^−2^, and the lowest at the Avlita and Pavlovsky Cape stations (~1.7 times). The minimum values for this parameter were found at the Sukharnaya (1.53 ± 0.31 ind.m^−2^) and Inkerman (2.57 ± 0.51 ind.m^−2^) stations.

It follows that there are four areas according to the distribution of ichthyoplankton depending on hydrometeorological parameters.

In the western district at the Ravelin station, the maximum egg abundance in the winds of the northern direction (with their repeatability of 20%) was noted. In the southern area of Sevastopol Bay, the situation is different at each station; where the temperature was higher, the concentration of ichthyoplankton was higher also. Attention to the close values for temperatures and numbers of ichthyoplankton under influence of winds from the south and south-west directions at the Pavlovsky Cape and Avlita stations were drawn. In the eastern area of the Bay (stations Sukharnaya and Inkerman), minimum values for ichthyoplankton abundance at lower temperatures and winds of the northern, western and north-western directions were observed.

### 3.2. Inter-Annual Variability in the Ichthyoplankton Number

**Figure 4:**
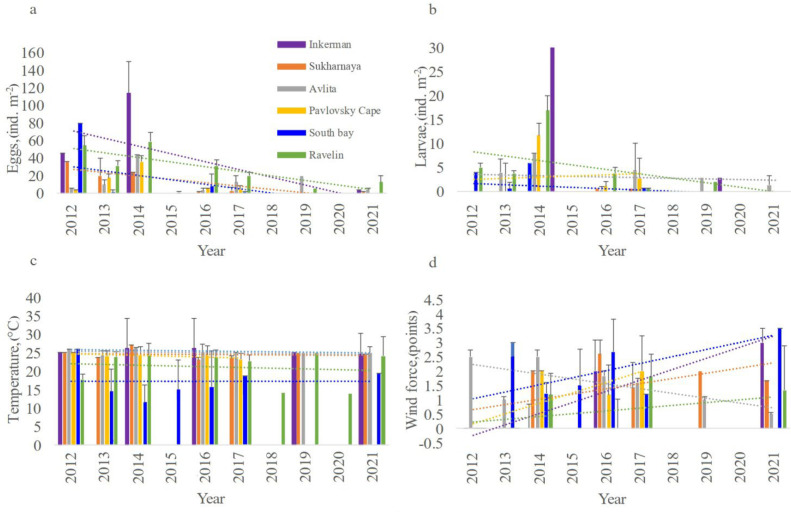
Inter-annual dynamics of the number of eggs (**a**) and larvae (**b**), temperature (**c**), wind strength (**d**) with standard deviations at the Inkerman, Sukharnaya, Avlita, Pavlovsky Cape, South Bay and Ravelin stations in Sevastopol Bay in summer 2012–2021. Dotted lines: linear trends.

We focused separately on the interannual variability of the ichthyoplankton abundance and hydrometeorological characteristics at the stations of the Sevastopol Bay in the summer of 2012–2021 (Figure 4). Increments of intra-decadal trends in the dynamics of ichthyoplankton abundance and hydrometeorological parameters at stations in Sevastopol Bay in summer 2012–2021 are reflected in Table 2.

In the study period, there is an increase and alternation in linear trends that is reported in Figure 4 and Table 2. The analysis of intra-decadal trends from 2012 to 2021, according to the available synchronous data, showed a general decrease in the number of eggs and larvae at all stations of the Sevastopol Bay with a general decrease in temperature, but under different meteorological conditions (different forces and directions of winds). The greatest decrease in water temperature occurred in the central and eastern regions of the Bay (by 1 °C), the lowest occurred in the western and southern parts (by 0.05 °C). In the western region of the Bay, there was an increase in the north and south-west winds, and in the eastern region, there was an increase in north, west and north-west winds. In the central part, there was a decrease in northerly winds (Figure 2, Table 2).

PCA and ANOVA analyses were performed to confirm intra-decadal trends and relationship between the ichthyoplankton abundance and the studied environmental parameters, and a correlation was also calculated using the Pearson correlation test (Table 3).

**Figure 5:**
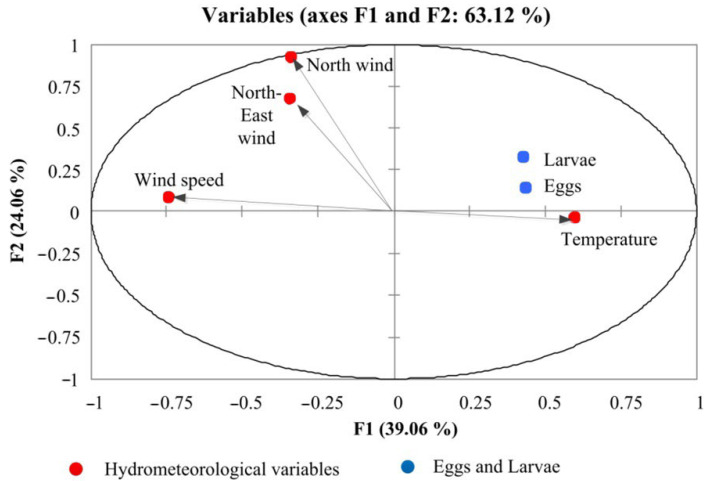
Correlation biplot diagram based on the principal component analysis (PCA) of the hydrometeorological variables and abundance of ichthyoplankton.

Based on the correlation analysis, a positive relationship was established between number of eggs and larvae and temperature (r > 0.82 and r > 0.84) and the repeatability of the northeasterly wind (r > 0.46 and r > 0.58), whereas a negative relationship emerged between the wind speed and the number of eggs (r < −0.60) and larvae (r < −0.49) in the South Bay. PCA analysis showed a positive effect of temperature and the northeasterly wind and a negative effect of the total wind speed of ichthyoplankton number. The factor diagram shows the cumulative effect of various environmental factors on the abundance of ichthyoplankton (Figure 5).

Temperature and winds cause 63.12% of the influence. ANOVA analysis confirmed the positive cumulative effect (*p* = 0.02) of temperature and wind from the north-east direction on the number of eggs and larvae in the South Bay.

Similar calculations of correlations and diagrams for the rest of the bay were performed. In the western, eastern and central parts of the bay, a positive relationship between the number of larvae (r = 0.40) and eggs (r = 0.5) and the repeatability of the north wind, as well as a negative relationship with its speed (r = −0.6) was established.

The PCA analysis revealed a positive effect of temperature and winds of northern directions, a negative effect of the east wind and the total wind speed on the ichthyoplankton number in all considered districts Sevastopol Bay.

## 4. Discussion

The analysis of the ichthyoplankton abundance in the summer period of 2012–2021 showed the heterogeneity of the spatial distribution in Sevastopol Bay across stations. We have identified four areas according to the distribution of the amount of ichthyoplankton: western (Ravelin), southern (Southern Bay), central (Avlita and Pavlovsky Cape) and eastern (Sukharnaya and Inkerman). According to the level of pollution of Sevastopol Bay, four areas were also distinguished [13,16,20]. It is known that the pollution of the water thickness of the southern part of Sevastopol Bay depends both on the intensity of the intake of pollutants and on the wind regime [16]. The significance of the wind regime for the southern region is confirmed by the work on modeling the pollution spread in the Sevastopol Bay [21]. Numerical experiments on the contaminant spread from a possible release site in the central part (Gollandiya Bay, near Avlita) showed that the dependence of the direction/trajectory of the pollution spot on the type of water circulation was most evident in the Southern Bay, and to a lesser extent in the central part of Sevastopol Bay. Polluted water masses are retained in the southern part of the Bay with a predominance of northern winds; they are carried out with southern winds to the western part of Sevastopol Bay, affecting its ecological state [16]. This is consistent with the results of this work, since with the dominance of northern winds at one of the most polluted stations, the northern part of the Southern Bay, a fairly large average number of ichthyoplankton in 2012–2021 was recorded. In the southern region during the same period, ANOVA confirmed the positive cumulative effect (*p* = 0.02) of temperature and wind from the north-east direction on the number of eggs and larvae, and Pearson’s correlation analysis showed significant correlations between these parameters on an interannual scale (r > 0.5).

With northerly winds, high values for the number of eggs and larvae at Pavlovsky Cape and Ravelin stations were generally observed, which confirm the locking of pollution in the southern part of Southern Bay without spreading it along Sevastopol Bay. At the same time, the north-coast wind leads to coastal upwelling near Sevastopol [22], that is, the rise of nutrient-rich cool waters from deep layers to the surface. The water masses enriched with nutrients, under the influence of the already changed south-westerly wind direction (repeatability of 15%), can enter Sevastopol Bay. Thus, the egg abundance at Ravelin station is largely due to the effects of the superposition of winds from the north and south-west directions.

In the central part of the Bay, the picture is somewhat different. The fact is that in the northern part of the Sevastopol Bay, the Mackenzie Mountains approach it, and from the south, the Balaklava Heights. The height of the coastal ledges in the area of Avlita station and the central part is about 200 m above sea level. These coastal ledges block the north wind. Its influence weakens in the central part and creates more favorable conditions for the survival of species. Thus, the Avlita station, located in the central region, is an exception, where the orography of the northern coast, contributing to the weakening of the north wind, has a significant impact on the number of ichthyoplankton.

The complex pattern of the coastline of the bay and its significant area affect the distribution of ichthyoplankton. For example, it is known that the distribution of pelagic fish species eggs and larvae is limited from the exit from Sevastopol Bay to the Southern Bay. Pelagic fish practically do not go deep into the Sevastopol Bay, and this fact can influence by way of a decrease in the number of ichthyoplankton as far from the entrance to the bay [3]. Our results allow to clarify the minima of the eggs and larvae number in the eastern part (Inkerman and Sukharnaya stations). This may also be due to the influence of northern, western and north-westerly winds driving ichthyoplankton into the eastern part of the Bay. Due to the fact that the eastern end of the Bay is the mouth of the Chernaya River, an additional factor of negative impact may be pollution by domestic wastewater of the river [23,24,25]. Thus, our results confirm both the importance of wind influence in the pollution of Southern Bay, as shown by other researchers, and reveal the importance of this factor in other areas of Sevastopol Bay.

Another factor affecting the ichthyoplankton number is the river runoff nutrients. In general, there was an increase in nitrate and phosphate in Sevastopol Bay in 2012–2016, mainly in the winter–spring period [15], which is also associated with winter maxima up to 50% of precipitation. The Mediterranean type of climate determines the seasonal nature of precipitation distribution. During the dry summer period, one of tributaries Aitodorka is still of sufficient importance, since it is fed by springs, and the other Sychaya River, fed by rainwater, dries up. Thus, there is low water in the Chernaya River and practically no precipitation—15–20%, on which the amount of nutrients entering the Bay depends and this factor does not have a significant impact. This fact can explain the minimum values for the ichthyoplankton number at the Sukharnaya and Inkerman stations in the eastern district of Sevastopol Bay. In general, there is a direct relationship between the summer-spawning ichthyoplankton abundance in all the parts of the Bay on temperature, which is consistent with the results from various researchers [2,26]. At lower summer temperatures, the minimum values for the number of eggs and larvae were observed, at the highest temperatures, the greatest values were observed.

Analysis of the intra-decadal trends for 2012–2021 showed a general decrease in the ichthyoplankton number throughout the bay. A similar trend in the coast of Sevastopol was found [27]. Thus, the Sevastopol Bay is losing its fishing and economic importance, as previously noted by various researchers [3,5].

We have shown the identified trends are associated not only with the increasing anthropogenic impact on the ecosystem of Sevastopol Bay, but also with the peculiarities of orography, temperature and wind regime. The effect of all these factors on an intra-decadal scale negatively affects the ecological status and recreational attractiveness of Sevastopol Bay.

## 5. Conclusions

Analysis of the decadal variability in the number and spatial distribution of eggs and larvae allowed us to identify four areas in Sevastopol Bay: western (Ravelin), southern (Southern Bay), central (Avlita, Pavlovsky Cape) and eastern (Sukharnaya, Inkerman).

The intra-annual variability of the number of ichthyoplankton is characterized by the maxima of eggs during the spawning period (July–August), and minima (March–April) for the period 2012–2021.

The trend of a general decrease in the number of ichthyoplankton at all stations with an increase in north and north-east winds and temperature decrease for the period 2012–2021 was found.

During the summer spawning in all areas of Sevastopol Bay, PCA showed a positive effect of temperature and repeatability of winds of northern and northern-eastern directions and a negative effect of wind speed.

Wind regime, spread of pollution, biogenic elements and river runoff affect the abundance of summer spawning ichthyoplankton in Sevastopol Bay in 2012–2021.

## Figures and Tables

**Table 1:** Average (±SD) values for ichthyoplankton abundance and hydrometeorological characteristics and their standard deviations in the Sevastopol Bay in the summer of 2012–2021.

Stations Name	Parts	Eggs, (ind.m^−2^)	Larvae, (ind.m^−2^)	T, (°C)	Wind Force, (Points)	Wind Direction
Ravelin	West part (4)	24.74 ± 4.95	3.85 ± 0.77	24.14 ± 2.27	1.75 ± 0.95	N, SW
SouthBay	South part (5)	21.83 ± 4.37	11.04 ± 2.21	25.42 ± 1.87	2.17 ± 0.75	N, NW, SW
Pavlovsky Cape	Central part (3, 4)	20.32 ± 4.06	7.99 ± 1.60	24.44 ± 1.79	1.69 ± 0.84	S, SW
Avlita		20.28 ± 4.50	6.40 ± 2.53	24.44 ± 4.94	1.42 ± 0.28	SW, N
Suharnaya	East part (1, 2)	13.17 ± 2.63	1.53 ± 0.31	23.75 ± 2.00	2.29 ± 0.76	N, W
Inkerman		16.06 ± 3.21	2.57 ± 0.51	24.63 ± 1.34	2.33 ± 1.15	N, NW

**Table 2:** Increments of intra-decadal trends in the dynamics of ichthyoplankton abundance and hydrometeorological parameters at stations in Sevastopol Bay in summer 2012–2021.

Stations Name	Parts	Eggs, ind.m^−2^	Larvae, ind.m^−2^	T, °C	Wind Force, Points	Wind Direction
Ravelin	West part	**–40**	−8	−0.05	+1	N, SW
South Bay	South part	**–30**	−2	−0.05	**+2**	N, NW, SW
Pavlovsky Cape	Central part	−12	**−2**	−0.05	+1.5	S, SW
Avlita		−7.5	−1	−1.00	**–1.0**	N, SW
Suharnaya	East part	**–30**	−1.5	−0.50	+1.8	N, W
Inkerman		**–20**	−1	−0.80	**+3**	N, NW

Note: throughout Table 3, the “−” sign indicates negative trends (decrease), the ”+” sign indicates positive (increase) for the observation period under consideration. Trend increments with determination coefficients greater than 0.4 are highlighted in bold.

**Table 3:** Correlation matrix (Pearson (n−1)).

Variables	T	NE Wind Repeatbility	Wind Speed	Eggs	Larvae
T	**1**				
NE wind repeatbility	0.22	**1**			
Wind speed	−0.38	0.21	**1**		
Eggs	**0.82**	**0.46**	**−0.60**	**1**	
Larvae	**0.84**	**0.58**	**−0.49**	**0.97**	**1**

Values in bold are different from 0 with a significance level alpha = 0.05.

## Data Availability

All data included in this study are available upon request by contact with the corresponding author.

## References

[1] Hjort J. (1914). Fluctuations in the Great Fisheries of Northern Europe Viewed in the Light of Biological Research.

[2] Dekhnik T.V., Pavlovskaya R.M. (1979). Dynamics of number, survival and elimination of eggs and larvae of mass fish. Foundations of Biological Productivity of the Black Sea.

[3] Gordina A.D., Klimova T.N. (1993). Ichthyoplankton of Sevastopol bays. Ichthyofauna of the Black Sea Bays in the Conditions of Anthropogenic Impact.

[4] Gordina A., Pavlova E., Ovsyany E., Wilson J., Kemp R., Romanov A. (2001). Long-term changes in Sevastopol Bay (the Black Sea) with particular reference to the ichthyoplankton and zooplankton. Estuar. Coast. Shelf Sci..

[5] Gordina A.D., Tkach A.V., Pavlova E.V., Klimova T.N., Ovsyanyi E.I., Romanov A.S. (2003). The state of ichthyoplankton communities in the Sevastopol Bay (Crimea) in May–September 1998 and 1999. Probl. Ichthyol..

[6] Gordina A.D., Salekhova L.P., Klimova T.N. (2004). Fish Species composition as an indicator of the current state of the coastal ecosystem of the South-Western shelf of the Crimea. Mor. Ekol. Zhurnal.

[7] Kuftarkova E.A., Rodionova N.Y., Gubanov V.I., Bobko N.I. (2008). Hydrochemical characteristics of individual bays of the Sevastopol seashore. Works YugNIRO.

[8] Klimova T.N., Vdodovich I.V., Ignatiev S.M., Seregin S.A., Kuzminova N.S. (2017). The Ichthyoplankton State in the Sevastopol Bay Mouth (Black Sea). J. Sib. Fed. Univ. Biol..

[9] Klimova T.N., Podrezova P.S. Species diversity of ichthyoplankton in areas of the coastal waters of Sevastopol with different degrees of anthropogenic load. Proceedings of the V International Scientific Conference of Aquatic Bioresources, Aquaculture and Ecology of Reservoirs.

[10] Belokopytov V.N., Lomakin P.D., Subbotin A.A., Shchurov S.V. (2002). Background characteristics and seasonal variability of the vertical stratification of the thermohaline field off the coast of Sevastopol. Ecol. Saf. Coast. Shelf Zones.

[11] Repetin L.N., Gordina A.D., Pavlova E.V., Romanov A.S., Ovsyany E.I. (2003). Influence of oceanographic factors on the ecological state of the Sevastopol Bay (Black Sea). Mar. Hydrophys. J..

[12] Mikhailova E.N., Shapiro N.B. (2005). Modeling of circulation and spatial structure of thermohaline fields in the Sevastopol Bay taking into account real external data (winter 1997). Mar. Hydrophys. J..

[13] Ivanov V.A., Ovsyany E.I., Repetin L.N., Romanov A.S., Ignatyeva O.G. (2006). Hydrological and Hydrochemical Regime of the Sebastopol Bay and Its Changing under Influence of Climatic and Anthropogenic Factors.

[14] Egorov V.N., Gulin S.B., Malakhova L.V., Mirzoeva N.Y., Popovichev V.N., Tereshchenko N.N., Gulina L.V. (2018). Rating water quality in Sevastopol Bay by the fluxes of pollutant deposition in bottom sediments. Water Resour..

[15] Orekhova N.A., Varenik A.V. (2018). Current hydrochemical regime of the Sevastopol Bay. Phys. Oceanogr..

[16] Sovga E.E., Mezentseva I.V., Slepchuk K.A. (2020). Comparison of assimilation capacity andtrophic index of various parts of the water area of the Sevastopol Bay. Ecol. Saf. Coast. Shelf Zones Sea.

[17] Kopytov Y.P., Minkina N.I., Samyshev E.Z. (2010). The level of pollution of water and bottom sediments of the Sevastopol Bay (Black Sea). Environ. Control. Syst..

[18] Müller P.H., Neuman P., Storm R. (1979). Tafeln der Mathematischen Statistik.

[19] Thode H.C. (2002). Testing for Normality.

[20] Egorov V.N. (2019). Theory of Radioisotope and Chemical Homeostasis of Marine Ecosystems.

[21] Belokopytov V.N., Kubryakov A.I., Pryakhina S.F. (2019). Modelling of water pollution propagation in the Sevastopol Bay. Phys. Oceanogr..

[22] Ivanov V.A., Mikhailova É.N., Shapiro N.B. (2008). Modeling of wind upwellings on the northwest shelf of the Black Sea near local features of the bottom topography. Phys. Oceanogr..

[23] Ovsyany E.I., Romanov A.S., Ignatieva O.G. (2003). Distribution of heavy metals in the surface layer of bottom sediments of the Sevastopol Bay (Black Sea). Mar. Ecol. J..

[24] Lopukhin A.S., Romanov A.S., Ovsyany E.I. (2007). Seasonal features of the hydrological and hydrochemical structure of the waters of the Sevastopol Bay, microplankton and the distribution of its biochemical components (Black Sea, observations 2004–2005). Ecol. Saf. Coast. Shelf Zones.

[25] Ablyazov E.R. (2016). Influence of interannual variability of salinity and water temperature on the structure of the Ichthyocen of the estuarine zone of the Chernaya River. Environ. Saf. Coast. Offshore Zones Sea.

[26] Dekhnik T.V. (1973). Ichthyoplankton of the Black Sea.

[27] Basova M., Krasheninnikova S., Fazio F. (2021). The long-term ichthyoplankton abundance summer trends in the coastal waters of the Black Sea under conditions of hydrometeorological changes. Estuar. Coast. Shelf Sci..

